# Successful Debulking of Tricuspid Valve Vegetation in Infective Endocarditis Using the Latest Computer-Assisted Vacuum Thrombectomy Technology

**DOI:** 10.7759/cureus.89728

**Published:** 2025-08-10

**Authors:** Nur Mando, Lillian Short, Bola Habeb, Asaad Mouselli, Otto Valdes, Rohit Amin, Daniel Antonious

**Affiliations:** 1 Internal Medicine, University of Florida College of Medicine/Ascension Sacred Heart, Pensacola, USA; 2 Cardiology, Ascension Sacred Heart, Pensacola, USA

**Keywords:** computer-assisted vacuum thrombectomy, infective endocarditis, penumbra lightning flash 2.0, tricuspid valve vegetation, valvular endocarditis

## Abstract

The incidence of infective endocarditis (IE) has risen globally, with intravenous drug use contributing significantly to its increasing prevalence. IE can lead to severe complications, including embolic stroke, heart failure, and septic embolization, often resulting in high morbidity and mortality. While traditional treatment involves long-term antibiotics and surgery, some patients are poor surgical candidates and require alternative interventions. This case report presents a 50-year-old woman who developed methicillin-resistant *Staphylococcus aureus* tricuspid valve endocarditis complicated by persistent bacteremia and septic emboli. Due to her inoperability, she underwent successful percutaneous mechanical thrombectomy using the Penumbra Lightning Flash 2.0 system (Penumbra, Inc., Alameda, CA). The Penumbra Lightning Flash 2.0 device offers a less invasive, effective method for debulking endocarditis vegetations, reducing procedural risks, such as blood loss, and requiring fewer resources than other thrombectomy devices like the AngioVac system. This case demonstrates the potential of the Lightning Flash 2.0 as a safer, more accessible treatment option for patients with difficult-to-manage IE who cannot undergo surgery.

## Introduction

The incidence of infective endocarditis (IE) has risen globally over the past decades, and the United States has become the country with the highest incidence at a rate of 15 per 100,000 people due to increasing rates of intravenous drug use (IVDU) [1]. Patients who are affected by endocarditis can have devastating effects such as embolic stroke, intracerebral hemorrhage, bacteremia, septic embolization resulting in organ infarct, heart failure, systemic immune reaction, osteomyelitis, and septic arthritis [2]. Due to its many complications, endocarditis is associated with high morbidity and mortality without appropriate treatment. Early diagnosis is imperative to improve outcomes, as is early surgical/procedural intervention in certain patients [2]. In patients with IE who require surgical intervention but are not good surgical candidates (i.e., patients who are considered a high surgical risk), less invasive methods such as mechanical thrombectomy are being used as an alternative method to remove or debulk vegetations. Surgical inoperability should include an assessment of hemodynamic instability or cardiogenic shock, renal failure, and surgery before completion of antibiotic therapy [2]. Here, we highlight a case report of an inoperable patient with methicillin-resistant *Staphylococcus aureus* tricuspid valve endocarditis who underwent successful tricuspid valve vegetation debulking with the Penumbra Lightning Flash 2.0 (Penumbra, Inc., Alameda, CA).

## Case presentation

**Figure 1:**
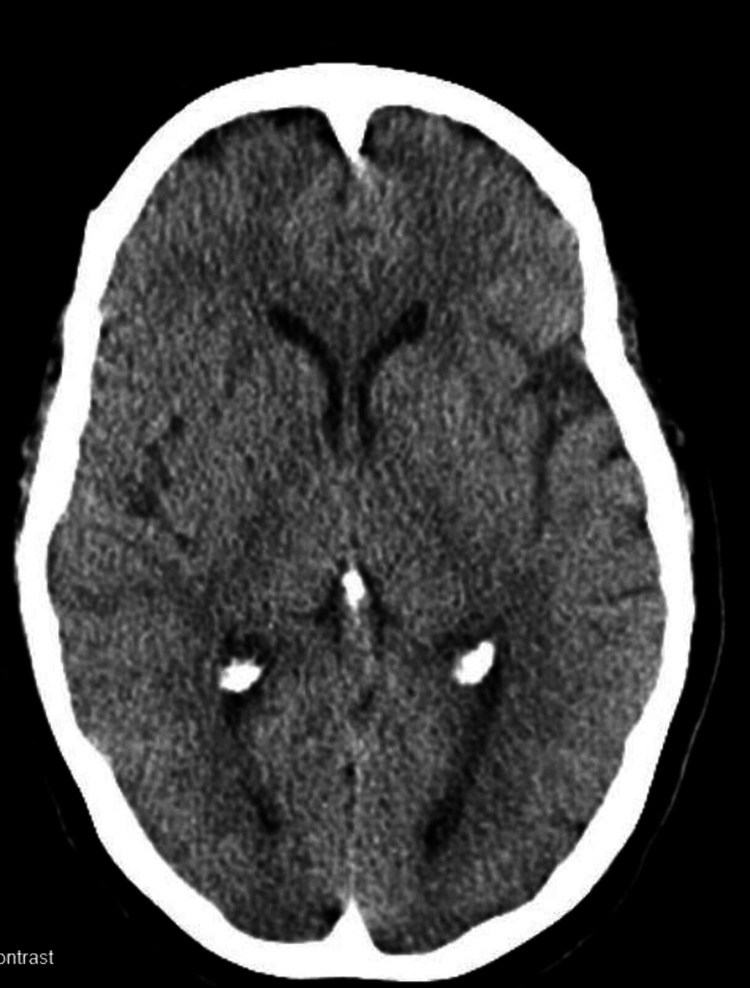
A CT of the head without contrast revealed no acute intracranial abnormalities.

**Figure 2:**
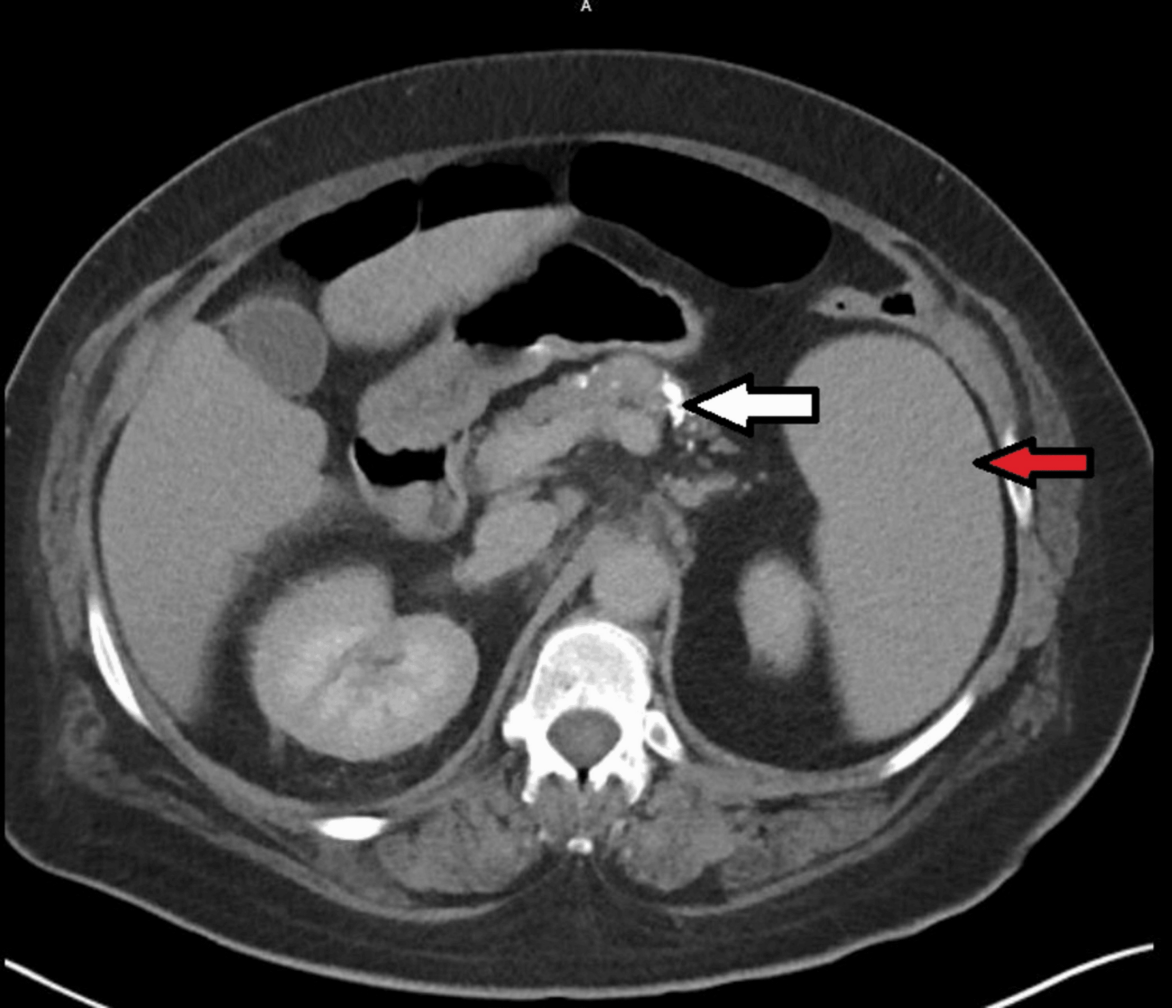
CT of the abdomen and pelvis with contrast revealed chronic pancreatitis (white arrow) and splenomegaly (red arrow).

**Figure 3:**
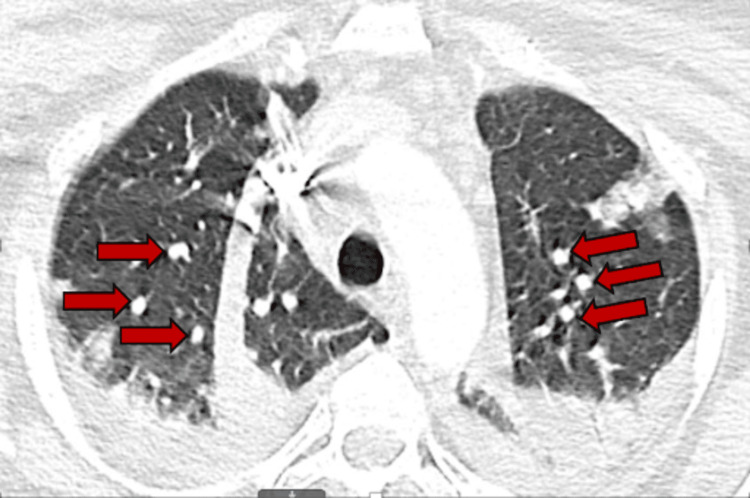
Computed tomography angiography revealed bilateral multifocal nodular infiltrates concerning for septic emboli.

A 50-year-old Caucasian female with non-insulin-dependent type II diabetes mellitus, obesity, hypothyroidism, osteoarthritis, and IV drug abuse presented to the emergency department (ED) with a chief complaint of left upper quadrant abdominal pain, nausea, and vomiting for three days. She denied any known cardiac surgery or medical history. Upon arrival to the ED, the patient met severe sepsis criteria with fever, tachycardia, tachypnea, leukocytosis, and multifocal pneumonia/cellulitis as a suspected source of infection. Her lactic acid was elevated at 2.9, and her white blood cell (WBC) count was 20 (Table 1). Her thyroid-stimulating hormone was within normal limits. The patient initially received empiric antibiotic treatment with intravenous (IV) Zosyn. An indwelling Foley catheter was placed in the ED on arrival, and a urinalysis was obtained, which revealed moderate blood: >1000 mg/dL glucose, 100 protein mg/dL, >80 ketones mg/dL, and 5-10 hyaline casts. A urine drug screen was positive for methamphetamines. The patient admitted to parenteral and non-parenteral illicit drug use. Computed tomography (CT) of the head without contrast was negative for acute intracranial abnormalities (Figure 1). A CT of the abdomen and pelvis with contrast revealed splenomegaly and chronic pancreatitis (Figure 2). A CT angiography (CTA) of the chest ruled out pulmonary embolism but revealed bilateral nodular infiltrates concerning for septic emboli (Figure 3). On the first day of admission, two out of two blood cultures grew methicillin-resistant *Staphylococcus aureus* (MRSA), and the patient's antibiotic coverage was broadened to include IV vancomycin.

**Table 1:** Admission laboratory tests.

Parameter	Day 1	Reference range
Red blood cells	4.38	3.8-5.2 million cells/μL
Hemoglobin	11.7	12.0-15.4 g/dL
Hematocrit	35.0	35-45%
Mean corpuscular volume	79.9	81-98 fL
White blood count	20.7	4.0-11.0 cells/μL
Neutrophils	90.7	40-70%
Lymphocytes	4.1	20-45%
Eosinophils	N/A	1-6%
Monocytes	4.3	2-10%
Basophils	0.1	0-1%
Platelet count	245	150-400 cells/μL
Partial thromboplastin time	38.8	23-40 seconds
Prothrombin time	15.7	11.6-15.0 seconds
International normalized ratio	1.3	0-1.0
Lactic acid	2.9	0.5-2.2 mmol/L
Lipase	250	8-78 IU/L
Thyroid-stimulating hormone	0.356	0.350-4.940 mcIU/mL

On physical examination, the patient was obese. She had dry mucous membranes with poor dentition. She was hypovolemic. The patient was tachypneic, but her lungs were clear to auscultation bilaterally without wheezing, rhonchi, or rales. She was tachycardic, but she was without murmurs, gallops, or rubs upon auscultation.

On day two of admission, a transthoracic echocardiogram (TTE) was obtained, and an infectious disease (ID) specialist was consulted. The TTE revealed trace mitral valve thickening with mild aortic valve calcification, trace pulmonic regurgitation, and trace tricuspid regurgitation. The patient’s rheumatoid factor was elevated, and complement levels were low, indicating an autoimmune reaction. The patient had persistent MRSA bacteremia despite IV vancomycin, and the ID specialist transitioned the patient to IV daptomycin. Unfortunately, the patient was persistently bacteremic with MRSA.

We used the 2023 Duke-International Society for Cardiovascular Infectious Diseases (ISCVID) criteria for IE. The patient had a microbiologic major criterion, given that she grew a microorganism that commonly causes IE (MRSA) isolated from two or more separate blood culture sets. She also met several minor criteria, including injection drug use, fever with a temperature >38°C, and immunologic and vascular phenomena, including positive rheumatoid factor and septic pulmonary infarcts. This clinical picture indicated a diagnostic result of "definite endocarditis."

Due to increased clinical suspicion for IE with a Duke's criteria indicating definite endocarditis, cardiology was consulted to perform a transesophageal echocardiogram (TEE). However, the patient's respiratory status declined, and she required bilevel-positive airway pressure (BiPAP) and was transferred to the intensive care unit (ICU) for closer monitoring. She was hypotensive and required pressor support with norepinephrine.

**Figure 4:**
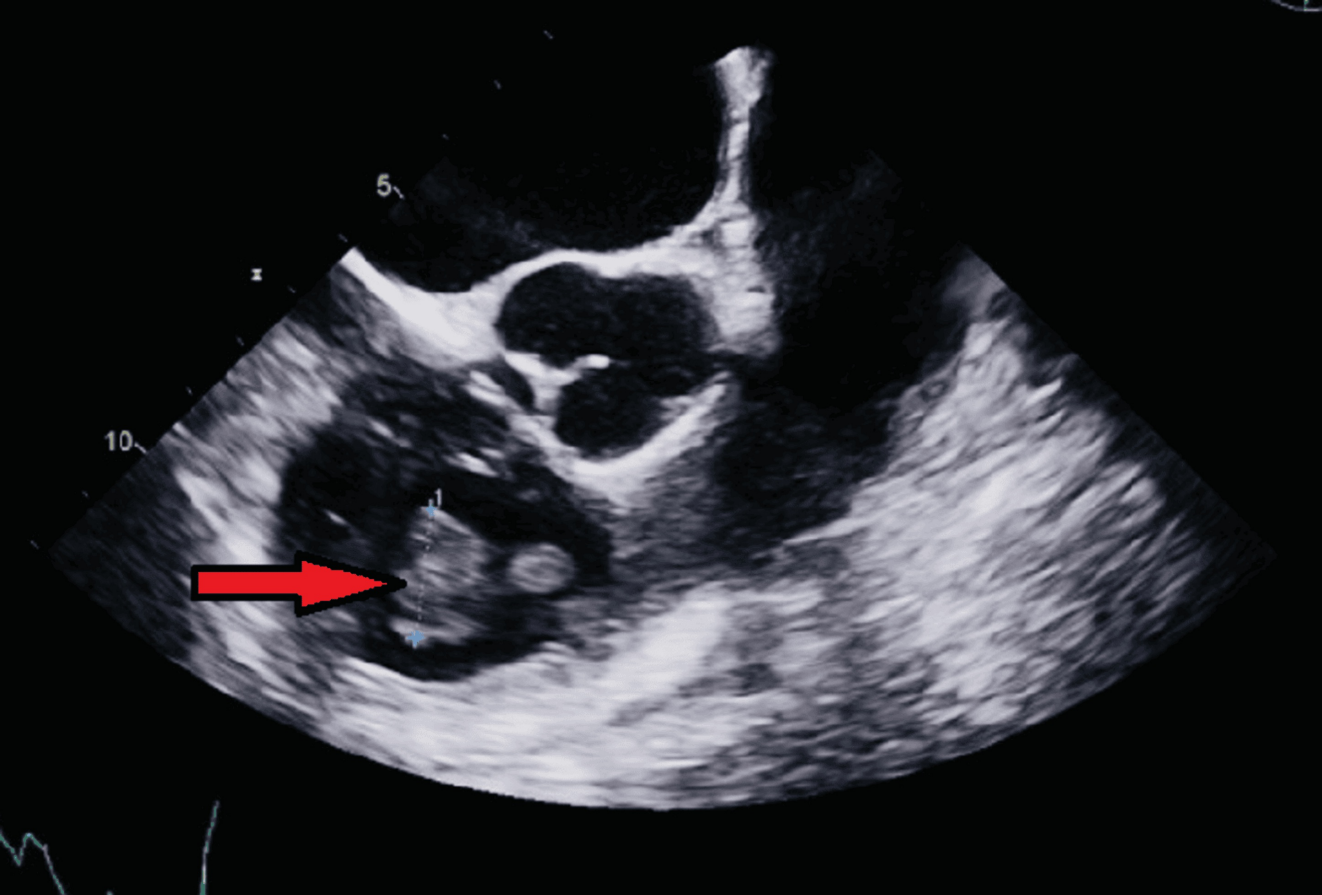
Transthoracic echocardiogram revealed a tricuspid valve vegetation extending into the proximal right ventricular outflow tract measuring 1.9 cm.

On day eight, a TEE was completed, which revealed a 19 mm tricuspid valve mobile, complex mass adherent to the chordal structures or papillary muscle of the tricuspid valve extending into the proximal right ventricular outflow tract (Figure 4). Cardiothoracic surgery was consulted, and the patient was deemed to be a poor surgical candidate due to her multiple co-morbidities, including diabetes mellitus, morbid obesity, and illicit drug use in conjunction with acute hypoxic respiratory failure and hemodynamic instability. Furthermore, the size of the valvular vegetation did not quite meet the guidelines for surgical intervention of right-sided vegetation of 20 mm. They recommended percutaneous mechanical aspiration with interventional cardiology instead.

**Figure 5:**
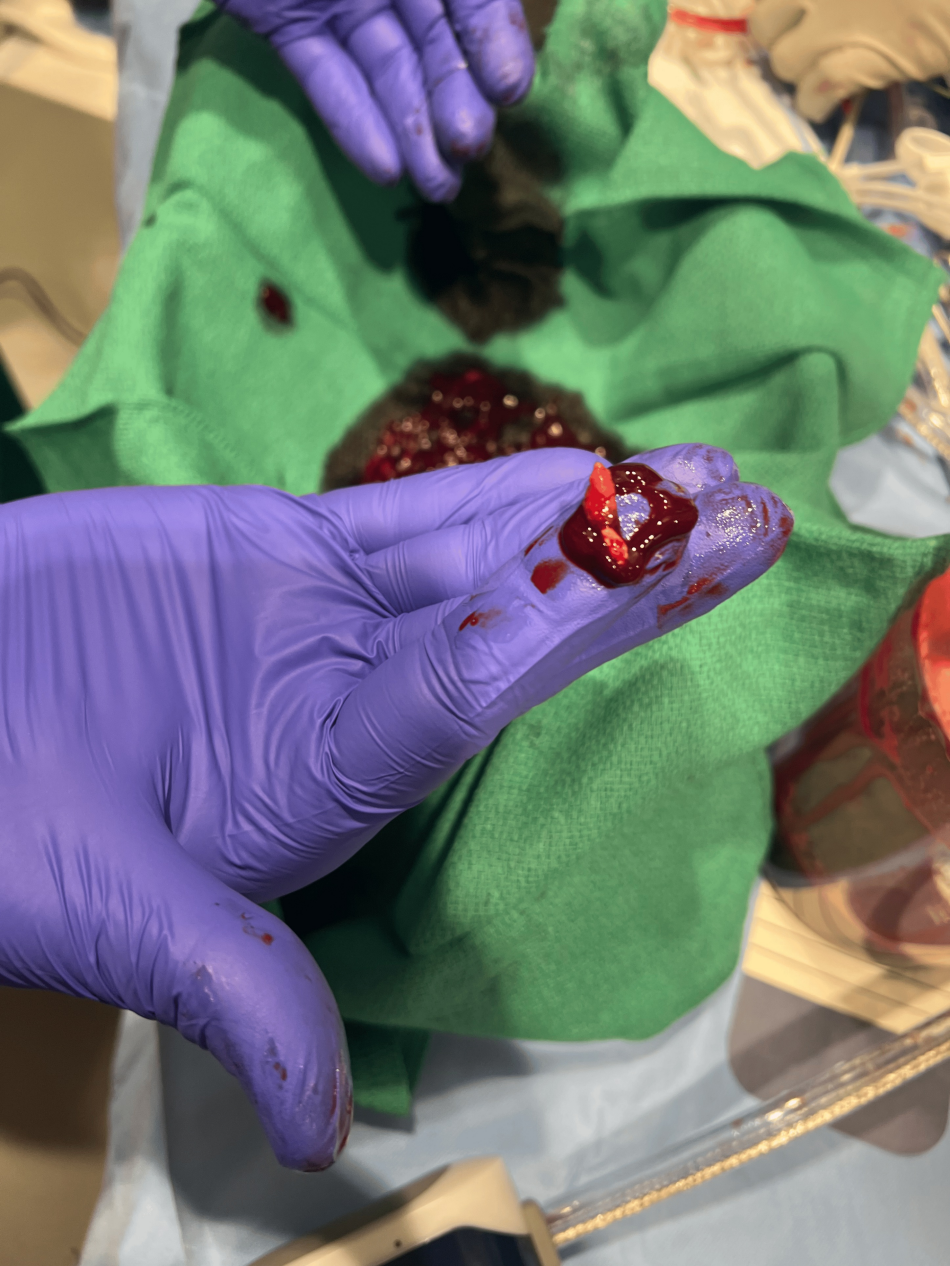
Aspiration of tricuspid valve vegetation using the Penumbra Lightning Flash 2.0.

On day nine, the patient underwent successful TEE-guided percutaneous mechanical aspiration using the Penumbra Lightning Flash 2.0 (Figure 5) and had significant vegetation debulking with trace tricuspid valve regurgitation improved compared with preprocedural imaging. There were no procedural complications. After debulking the tricuspid valve vegetation, blood cultures stopped growing MRSA, and she clinically improved on antibiotics. She was successfully transitioned out of the ICU and discharged home with recommendations by ID to continue IV daptomycin 750 mg/d for six weeks. She was instructed to follow up with her primary care physician, cardiology, infectious disease, and pulmonology. Unfortunately, the patient was lost to follow-up thereafter.

## Discussion

Endocarditis can be a complex disease to diagnose and can thus cause delays in initiation of treatment, which can cause poor patient outcomes. The clinical presentation of endocarditis can be very diverse, ranging from an indolent low-grade febrile illness to multiorgan failure and death [2]. There must be a high clinical suspicion of IE, and an official diagnosis of IE can be made utilizing the 2023 Duke-ISCVID criteria.

Endocarditis is typically caused by gram-positive streptococci, staphylococci, and enterococci infections. Together, these three groups account for 80% to 90% of all cases [3]. Specifically, in IVDU-related endocarditis, *Staphylococcus aureus* is the most common culprit (60-90%). In addition to various streptococci species, other common colonizers of the oropharynx, such as the HACEK organisms (*Haemophilus*, *Actinobacillus*, *Cardiobacterium*, *Eikenella*, and *Kingella*), can less frequently be the culprit bacteria [3]. Typically, patients present with community-acquired endocarditis due to immunosuppression, IVDU, poor dentition, degenerative valve disease, and rheumatic heart disease. IVDU, which underlies almost 10% of infectious endocarditis cases, suggests repeated inoculation with skin flora such as *S. aureus* and *S. epidermidis*, with *S. aureus* demonstrating a predilection for healthy, native tricuspid valves [3].

Once diagnosed, stable patients typically require IV antibiotics and/or a combination of oral antibiotics for up to six weeks [4]. However, select patients require early surgical treatment. Surgical intervention is performed before an entire course of antibiotics is completed. Early surgical treatment depends on clinical and prognostic factors. In left-sided endocarditis, indications for surgery include highly resistant organisms, evidence of abscess or penetrating lesion, persistent bacteremia, left-sided mobile vegetations >10 mm without respect to lesion severity or operative risk (class IIb), those with prosthetic valve endocarditis with relapsing infection, and acute, severe left-sided valve regurgitation regardless of the New York Heart Association (NYHA) class [5]. Indications for right-sided surgical intervention include right heart failure secondary to severe tricuspid regurgitation with poor response to medical therapy, vegetations >20 mm, recurrent septic pulmonary emboli, presence of a highly resistant organism, and persistent bacteremia despite antimicrobial therapy [6].

If a patient is deemed to be a poor surgical candidate but needs further treatment in addition to IV/oral antibiotics, therein lies a gap in treatment. As an alternative treatment, aspiration devices have been used to de-bulk vegetations seen in IE without the need for surgery. Aspiration devices are medical tools designed to remove undesired material such as thrombi, emboli, vegetations, or other intravascular debris. These specialized vacuum-assisted devices are designed to eliminate undesirable intravascular material such as thrombi, emboli, and rarely vegetations. While this technology offers a less invasive alternative for vegetation removal in select endocarditis patients, its use is generally reserved for cases where traditional surgical methods are contraindicated or pose significant risks [5]. The decision to employ this technique should be individualized, taking into consideration the patient's overall health status, the size and location of the vegetation, and the presence of any comorbid conditions. This concept of debulking tricuspid valve vegetation aims to reduce bacterial loads to allow antimicrobial therapy to cure the infection or stabilize the patient as a bridge to surgery [7].

Numerous other systems on the market have been previously used to debulk vegetations in endocarditis. The only FDA-approved device for endocarditis is the AngioVac system (AngioDynamics, Latham, NY). The AngioVac System is designed for extracorporeal aspiration and filtration of intravascular debris [8]. It is commonly used to retrieve large right-sided IE vegetations, thrombi in the right atrium, inferior vena cava (IVC), or pulmonary arteries. Other options include the FlowTriever System (Inari Medical, Irvine, CA), Aspirex Catheter (Straub Medical, Wangs, Switzerland), and Medtronic Export Aspiration Catheter (Medtronic, Dublin, Ireland). A comprehensive systematic review and meta-analysis of 301 patients from 44 studies investigated the efficacy and safety of the AngioVac device as an option for extracting right-sided IE vegetations. There was a demonstrated procedural and clinical success of 89.2% and 79.1%, respectively. In 90% of cases, there was a vegetation removal of >50%. Bacteremia clearance was achieved in 82.5% of patients [8]. There are no head-to-head studies between the AngioVac and Lightning Flash 2.0.

The AngioVac and the Lightning Flash 2.0 have sustained aspiration versus syringe-based aspiration for other devices. Sustained aspiration allows for the negative pressure of the catheter to be sustained and decreases procedure time. However, the Lightning Flash 2.0 device offers an advantage because the device has a unique "gallop" feature, in which intelligent clot-sensing technology can sense pressure and flow within the catheter. As such, the device can recognize the thrombus more effectively and only aspirate when the clot is truly engaged. This augments thrombus removal and allows for minimal blood loss during the procedure [9]. The AngioVac, on the other hand, does not have this “gallop” feature and instead utilizes an extracorporeal circuit where the AngioVac cannula is used as a filter to remove thrombi. While using an extracorporeal circuit allows for simultaneous retransfusion of the blood lost, which minimizes the patient’s blood loss, it does require an anesthesiologist and a perfusionist [10]. Therefore, the Lightning Flash 2.0, while off-label for the indication of vegetation retrieval, allows for a potentially safer and more accessible solution to more patients globally, especially in less-equipped facilities. The potential adverse events of using an aspiration device such as the Lightning Flash includes acute vessel occlusion, air embolism, allergic reaction and anaphylaxis from contrast media or device material, anemia, arrhythmia arteriovenous fistula, cardiac injury, cardiac perforation, cardiac tamponade, cardio-respiratory arrest, compartment syndrome, death, emboli, emergent surgery, foreign body embolization, hematoma or hemorrhage at access site, hemoptysis, hemorrhage, hypotension/hypertension, infarction leading to organ damage, infection, ischemia, myocardial infarction, neurological deficits, including stroke, pneumothorax, pseudoaneurysm, renal impairment, or acute renal failure from contrast media, residual thrombus due to inability to completely remove thrombus or control blood flow, respiratory failure, valvular damage, vessel spasm, thrombosis, dissection (intimal disruption), or perforation [10].

The Lighting Flash could be a superior choice, and it should be considered as an option for those who cannot undergo surgical interventions but desperately need treatment in addition to antibiotics for the resolution of their endocarditis. Further studies are needed to validate generalizability.

## Conclusions

The case demonstrates the successful utilization of the Penumbra Lightning Flash 2.0 device to improve outcomes for our patient with a significant vegetation burden who could not undergo traditional surgery. The Lightning Flash 2.0 device reduces procedural risks, including blood loss, by harnessing a less invasive approach. It can be implemented with fewer resources, making it more readily available to improve outcomes for more patients. This case report underscores that the Lightning Flash 2.0 may serve as a more widely available and safer first-line treatment for patients with difficult-to-manage IE.
